# Supply of opioids and information provided to patients after surgery in an Australian hospital: A cross-sectional study

**DOI:** 10.1177/0310057X231163890

**Published:** 2023-06-26

**Authors:** Ian SH Fong, Chin Hang Yiu, Matthew D Abelev, Sara Allaf, David A Begley, Bernadette A Bugeja, Kok Eng Khor, Joanne Rimington, Jonathan Penm

**Affiliations:** 1 Department of Pharmacy, Prince of Wales Hospital, Randwick, Australia; 2 School of Pharmacy, The University of Sydney Faculty of Medicine and Health, Camperdown, Australia; 3 Department of Pain Management. Prince of Wales Hospital, Randwick, Australia; 4 District Pharmacy Services, South Eastern Sydney Local Health District, Randwick, Australia

**Keywords:** Acute pain, opioids, surgery, hospital, discharge

## Abstract

Opioids are commonly prescribed to manage pain after surgery. However, excessive supply on discharge can increase patients’ risk of persistent opioid use and contribute to the reservoir of unused opioids in the community that may be misused. This study aimed to evaluate the use of opioids in Australian surgical patients after discharge and patient satisfaction with the provision of opioid information after discharge. This prospective cohort study was conducted at a tertiary referral and teaching hospital. Surgical patients were called 7–28 days after discharge to identify their opioid use and the information that they received after discharge. In total, 66 patients responded. Most patients underwent orthopaedic surgery (45.5%; 30/66). The median days of opioids supplied on discharge was 5 (IQR 3–5). In total, 40.9% (27/66) of patients had >50% of their opioids remaining. Patients undergoing orthopaedic surgery were less likely to have >50% of their opioids remaining (*P* = 0.045), whilst patients undergoing urological or renal surgeries were significantly more likely (*P* = 0.009). Most patients recalled receiving information about their opioids (89.4%; 59/66). However, the majority (51.5%; 34/66) did not recall receiving any information about the signs of opioid toxicity and interactions between opioids and alcohol. In conclusion, around 40% of patients had more than half of their opioid supply remaining after they ceased taking their opioid. Although most patients recalled receiving information about their opioids, more than half did not recall receiving any information about the signs of opioid toxicity or interactions between opioids and alcohol.

## Introduction

Opioid analgesics are commonly prescribed to manage pain after surgery.^1^ A study across Australia and New Zealand showed that around 59% (*n* = 1450) of surgical patients are prescribed opioids on discharge.^2^ Opioids are not expected to continue long term after surgery.^3^ However, a systematic review of 13 studies showed that persistent opioid use among surgical patients in Australia generally ranged from 3.9% to 10.5% at between two and four months after discharge.^4^ Even a couple weeks of opioid use after surgery may increase the risk of persistent opioid use, as Shah et al. showed that 24% of patients using opioids for 12 days continued to use opioids for one year.^5^ This proportion of patients with persistent opioid use increased to around 35% after using opioids for 21 days and then to around 43% after using opioids for 31 days.^5^ This risk of persistent opioid use is approximately doubled when a cumulative opioid dose exceeds 120 mg in oral morphine equivalent daily dose (oMEDD) during the starting month.^5–7^ Persistent opioid use can lead to changes in mood, sleep disturbances, increased risk of fractures, dependence, diversion, overdose and drug-related death.^1,8,9^ Therefore, it is important that patients are counselled on the appropriate use of opioids and not prescribed an excessive amount of opioids on discharge to minimise the risk of harm from persistent and accidental overuse of opioids by patients, families and the broader community.

To prevent persistent opioid use after surgery, researchers have evaluated how much opioids are supplied to patients on discharge and how they use their opioids after surgery. A previous Australian study (*n* = 1450) found that a median of 20 oxycodone tablets were given on discharge after surgery. Of these patients, 70% had leftover opioids at two weeks following discharge.^10^ These data are similar to international studies that reported 71–83% of surgical patients ceased their opioids due to adequate pain control.^11,12^ These international studies also highlighted poor understanding of the risks of opioids by patients, with only 4–30% of those with leftover opioids planning to dispose of them^11–13^ and only 23–27% storing them in a locked container or area.^11^ Unused opioids that are not safely disposed of or stored appropriately can cause harm to the community by potentially contributing to non-medical use of opioids and secondary exposure to family members and friends.^11–13^

Due to the risks of opioids and unsafe storage and disposal, patients should be provided with information about their opioids before discharge. Currently, it is unknown how much information patients receive about opioids on discharge. However, a study by Stanley et al. showed that only 6.9% of patients are given an opioid tapering plan on their discharge summary prior to implementing opioid education interventions in a Victorian hospital.^14^ This suggests that little written information may be provided to patients and general practitioners about patients’ opioids.

Given the limited data on opioid use in Australian surgical patients after hospital discharge, we aimed to evaluate the use of opioids in Australian surgical patients and their satisfaction with the provision of opioid information within a month of discharge.

## Methods

### Study design and recruitment

This prospective observational cohort study was conducted at a tertiary referral and teaching hospital in Sydney, New South Wales. Approval was obtained from the South Eastern Sydney Local Health District Human Research Ethics Committee (LNR/18/POWH/142), and a site-specific approval was granted.

Recruitment took place over a three-month period, between 1 December 2019 and 28 February 2020. Eligible patients were identified from operating theatre lists and patient lists from the electronic medical record. Following their surgery and prior to discharge, these patients were approached by a member of the research team who was not directly related to their care, and written consent was obtained. Patients were also provided with a participant information sheet and questionnaire for the phone interview.

### Eligibility criteria

Patients were considered eligible to participate if they were aged ≥18 years, were able to converse in English, managed their own medicines at home, had undergone surgery and were prescribed opioids on discharge. Patients were excluded if they had significant cognitive impairment or acute mental illness that would affect their participation or were currently on opioid substitution therapy for opioid dependence.

### Data collection

Baseline data of eligible patients were collected at the time of discharge from the electronic medical record (Cerner PowerChart, Cerner, Kansas City, MO), including patient demographics, Charlson Comorbidity Index to describe the comorbidities in the patient population^15^ and type of surgery (minor or major, elective or emergency, and surgical specialty). History of opioid use was also obtained from the electronic medical records in the patients’ medical admission and pharmacist medication reconciliation. Major surgery included open thoracic or abdominal surgery, surgeries where organs were removed (cholecystectomy, appendectomy) and joint replacements. Other surgeries such as open reduction and internal fixations, arthroscopy and washouts were classified as minor surgeries. The amount of regular and ‘as needed’ opioids given to the patient prior to discharge was obtained from the medication administration record and converted to the oMEDD using Opioid Dose Equivalence Calculation Table developed by the Faculty of Pain Medicine, Australian and New Zealand College of Anaesthetists.^16^ The quantity of opioids provided on discharge was obtained from dispensing records in iPharmacy (CSC, Macquarie Park, Australia) and discharge summaries from the electronic medical record. Discharge summaries are the only communication GPs receive about patients’ opioid management plans from the hospital.

Any concurrently prescribed non-opioid analgesics, such as paracetamol, non-steroidal anti-inflammatory drugs (NSAIDs), amitriptyline, nortriptyline, duloxetine, venlafaxine, lidocaine, clonidine, baclofen, carbamazepine, gabapentin and pregabalin, were also recorded from the patients’ discharge summary. The discharge summaries were also reviewed for any documented plans regarding opioids, such as a recommendation to wean the dose or cease after a number of days, or for the general practitioner to review the patient’s pain. Refer to Supplemental Appendix A for the baseline data collection form.

Patients were followed up by phone 7–28 days after discharge to investigate their ongoing use of opioids using a structured interview guide. This interview guide included questions developed by the research team and questions adapted from Satisfaction with Information about Medicines Scale (SIMS; Supplemental Appendix B).^17^ The guide was pilot tested with three patients and five pharmacists before being finalised. The interview guide used was not tested for reliability or validity, except questions based on the SIMS.

At the time of recruitment, patients were informed that they would be called on a pre-specified date, as calls appeared as a private number from a hospital phone. If they did not answer, a voicemail was left, and they were contacted again at a different time and day. Up to two additional attempts were made before the patient was classified as a non-responder. Patients were asked questions about their current use of opioids, including who was involved in the decision to continue or stop taking opioids. Specific questions were asked to ascertain the remaining supply of opioids from the hospital, and if and how any remaining supply was disposed of. Patients were also asked whether their opioids and pain had been reviewed by a general practitioner. Providing an information sheet on safe storage, withdrawal and tapering of opioids is recommended but not required for all patients being dispensed an opioid on discharge.^18^ Therefore, participant satisfaction with the opioid information received at the time of discharge was evaluated, as it was not common practice for patients to receive written information about opioids unless requested. All participants were asked to rate their level of satisfaction on 12 items of information from ‘too much’ information provided to ‘none needed’. These items were adapted from the SIMS (Supplemental Appendix B).^17^

As this was an observational study, we aimed to recruit patients over a three-month period, and no formal sample size calculation was performed. Assuming a loss to follow-up of approximately 20%, we anticipated a total of 80 patients would be included to describe opioid use after discharge.^10^

### Data analysis

The data were entered and managed in REDCap. They were analysed using Microsoft Excel (Microsoft, Redmond, WA) and IBM SPSS Statistics for Windows v25 (IBM Corp., Armonk, NY). The median and interquartile ranges are reported for continuous variables. Surgical specialties were categorised according to body system and/or clinical unit. The supply of opioids in days was calculated by dividing the number of tablets supplied on discharge by the number of equivalent tablets (based on oMEDD) taken over the 24-hour period prior to discharge. If no opioids were used over the 24-hour period prior to discharge, the quantity was reported as seven days, as this was the maximum quantity that may be supplied by the pharmacy department on discharge.

Comparison by speciality for oMEDD use 24 hours prior to discharge, opioid supply at discharge and proportion of patients having >50% or opioids remaining were conducted using the Mann–Whitney U-test for continuous data and Fisher’s exact test for categorical data. Comparison between minor and major surgery and proportion of patients having >50% of opioids remaining was also conducted using Fisher’s exact test. To assess non-response bias, demographic data of responders compared to non-responders were analysed using Fisher’s exact test and Mann–Whitney U-test.

## Results

### Patient characteristics

In total, 86 patients consented to the study. However, 20 did not respond to follow-up. Of the 66 patients who responded, most patients underwent minor surgery (43.9%; 29/66), and orthopaedics was the most common surgical specialty (45.5%; 30/66). The majority of patients had not used an opioid for at least two weeks before their admission (81.8%; 54/66).

When assessing non-response bias, no significant differences were observed in sex, age, surgery type or specialty between those who responded and non-responders (Table 1). However, responders had a higher Charlson Comorbidity Index than non-responders (Table 1).

**Table 1.** Demographic data of participants and non-respondents.

	Participants (*N* = 66)	Non-respondents (*N* = 20)	*P*-value
Sex (male), *n* (%)	38 (57.6)	12 (60)	0.802
Age (years), median (IQR)	52 (34–65)	38 (25–59)	0.1
Charlson Comorbidity Index	2 (0–4)	0 (0–1)	0.006
Surgery type,^a^ *n* (%)			
Major, elective	15 (22.7)	3 (15.0)	0.546
Major, emergency	7 (10.6)	5 (25.0)	0.139
Minor, elective	15 (22.7)	7 (35.0)	0.380
Minor, emergency	29 (43.9)	5 (25.0)	0.192
Surgical specialty, *n* (%)			
Cardiothoracic	5 (7.6)	0 (0.0)	0.586
General/upper gastrointestinal, colorectal, oncological, acute	15 (22.7)	8 (40.0)	0.153
Neurosurgery	3 (4.5)	1 (5.0)	1.0
Orthopaedics	30 (45.5)	7 (35.0)	0.451
Plastics, ear nose throat	8 (12.1)	0 (0.0)	0.189
Urology, renal	5 (7.6)	4 (20.0)	0.203

^a^Major surgery included open thoracic or abdominal surgery, surgeries where organs were removed (laparoscopic bowel resection, cholecystectomy, appendectomy) and joint replacements (arthroplasty). Minor surgeries included operative fixation of bone fractures (open and closed), arthroscopy and joint or wound lavage and wound debridement.

IQR: interquartile range.

### Non-opioid analgesic supply on discharge

At discharge, 68.2% (45/66) of patients were prescribed regular paracetamol, and only 9.1% (6/66) were supplied paracetamol ‘as required’. Only 18.2% (12/66) were prescribed a regular NSAID on discharge (Table 2).

**Table 2.** Analgesics prescribed on discharge.

Patients (*N* = 66)	*n* (%)
Opioid frequency prescribed on discharge	
Opioid, regular only	2 (3.0)
Opioid, regular and ‘as required’	9 (13.6)
Opioid, ‘as required’ only	55 (83.3)
Opioid prescribed on discharge	
Oxycodone IR	48 (72.7)
Tapentadol IR	13 (19.7)
Tramadol IR	2 (3.0)
Paracetamol-codeine IR	1 (1.5)
Oxycodone-naloxone slow release	6 (9.1)
Tapentadol slow release	2 (3.0)
Non-opioid analgesics prescribed on discharge	
Paracetamol, regular	45 (68.2)
Paracetamol, ‘as required’	6 (9.1)
NSAID, regular^a^	12 (18.2)
NSAID, ‘as required’^b^	6 (9.1)
Other, regular^c^	9 (13.6)
Other, ‘as required’	0 (0.0)
Opioid use at least 7 days after discharge	
Still taking opioids	17 (25.8)
No longer using opioids	49 (74.2)
Used all supplied opioids	13 (29.7)
Disposed unused opioids in bin	12 (18.2)
Returned unused opioids to pharmacy	2 (3.0)
Did not dispose of unused opioids	22 (33.3)

^a^The regular NSAIDs prescribed were ibuprofen (*n* = 7; 10.6%) and celecoxib (*n* = 5; 7.6%).

^b^The ‘as required’ NSAID prescribed was ibuprofen.

^c^The other regular non-opioid analgesics were amitriptyline (*n* = 1; 1.5%), clonidine (*n* = 1; 1.5%), gabapentin (*n* = 2; 3%) and pregabalin (*n* = 7; 10.6%).

IR: immediate release; NSAID: non-steroidal anti-inflammatory drug.

### Opioid supply and management plan on discharge

The median days of opioids supplied on discharge was 5 (IQR 3–5), with no statistically significant difference between the specialties (Table 3). The median use of opioids 24 hours prior to discharge in oMEDD was 15 mg (IQR 7.5–46), with no statistically significant difference between the specialties (Table 3). In total, 21% (14/66) of patients receiving opioids on discharge had not required any opioids in the 24 hours prior to discharge.

**Table 3.** Opioid use and supply by specialty.

	oMEDD 24 hours prior to discharge, median (IQR)	*P*-value	Days supplied, median (IQR)	*P*-value	Patients with ≥50% opioids remaining, *n* (%)	*P*-value
All specialties (*N* = 66)	15 (7.5–46)	NA	5 (3–5)	NA	27 (40.9)	NA
Cardiothoracic (*N* = 5)	15 (0–75)	0.874	3 (2.5–5)	0.120	3 (60)	0.393
General/upper gastrointestinal, colorectal, oncological, acute (*N* = 15)	22.5 (7.5–30)	0.957	5 (3–5)	0.540	6 (40)	1
Neurosurgery (*N* = 3)	15 (7.5–165)	0.710	5 (5–7)	0.230	2 (66.7)	0.563
Orthopaedics (*N* = 30)	22.5 (5.6–69.4)	0.308	5 (3–5)	0.389	8 (26.7)	0.045
Plastics, ear nose throat (*N* = 8)	15 (0–36)	0.458	5 (3.5–7)	0.244	3 (37.5)	1
Urology, renal (*N* = 5)	15 (5–15)	0.222	5 (5–7)	0.078	5 (100)	0.009

IQR: interquartile range; NA: not applicable; oMEDD: oral morphine equivalent daily dose.

Only 24 (36.4%) patients had a documented opioid management plan in their discharge summary. Of these documented plans, most recommended that the general practitioner review the patient’s pain and opioid analgesia requirements (95.8%; 23/24). Some discharge summaries also included more specific recommendations such as tapering the dose (29.2%; 7/24) and ceasing opioids after a certain number of days (8.3%; 2/24). Neurosurgery included an opioid plan for all three of their patients. On the other hand, plastics, ear nose throat (ENT; *n* = 8) and urology/renal (*n* = 5) specialties did not include an opioid plan for any of their patients. The remaining specialties did not include an opioid plan in the discharge summary for most of their patients: 30 (45.5%) in orthopaedics, 15 (22.7%) in general/upper GI, colorectal, oncological, acute, and five (7.6%) in cardiothoracic surgery.

### Opioid use after discharge

After discharge, the majority of patients were no longer taking opioids (74.2%; 40/66). In total, 40.9% (27/66) of patients had >50% of opioids remaining. Of those, patients who had orthopaedic surgery were significantly less likely to have >50% of opioids remaining (26.7%; 8/30; *P* = 0.045), whilst patients who had urology or renal surgery were significantly more likely to have >50% of opioids remaining (100%; 5/5; *P* = 0.009). There was no significant difference between the proportion of patients with >50% of opioids remaining when comparing patients who had minor surgery to those who had major surgery (*P* = 0.122).

Oxycodone immediate release (IR) was the most common opioid used after discharge (72.7%; 48/66). Amongst the 48 patients discharged on oxycodone immediate release tablets, 33% (195/591) of total tablets were reportedly unused. Tapentadol IR was the second most common opioid used after discharge (19.7%; 13/66). Amongst the 13 patients discharged on tapentadol IR, 60.3% (85/141) of total tablets were reportedly unused.

Amongst the 21% (14/66) of patients who did not require any opioids in the 24 hours prior to discharge, the median number of opioid tablets remaining was 45% (IQR 7.5–45%), with 28.6% (4/14) of these patients not using any opioids after discharge. After discharge, 49 (74.2%) patients had seen their general practitioner, and 35 (71.4%) of these patients had had a discussion about pain management. Of these, 13/49 (26.5%) patients were given another prescription for opioids. Amongst these 13 patients, six (46.2%) had undergone orthopaedic surgery, and their median Charlson Comorbidity Index score was 0 (IQR 0–2).

### Opioid information provided on discharge

Most patients recalled receiving information about their opioids (89.4%; 59/66). Information was recalled as being provided by a pharmacist, a doctor, a nurse or a combination of healthcare professionals. Twenty-six (39.4%) received information from a pharmacist, 23 (34.8%) received information from a nurse and 19 (28.8%) received information from a doctor. Verbal education was the most common method (74.2%; 49/66), and some received written information (30.3%; 20/66). Of the patients who received either written or verbal information, 25 (42.4%) stated that they had to refer or think back to the information provided after being discharged from hospital.

**Figure 1.**
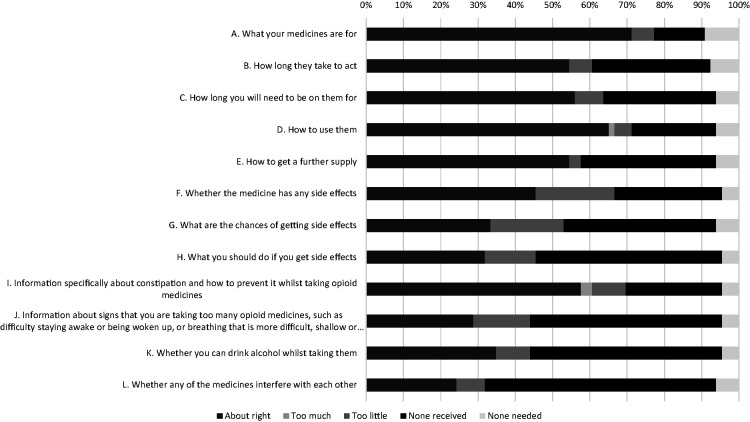
Satisfaction with different items of opioid information that patients recalled receiving on discharge.

Figure 1 shows the patient satisfaction with different items of information related to using opioids. In total, 32 (48.5%) patients reported that the information they received relating to how to take the opioid was ‘about right’ (items A, C and D; Supplemental Appendix B). However, 15 (22.7%) patients reported that they did not receive any information about side-effects (items F, G, H and I; Supplemental Appendix B). Furthermore, the majority (51.5%; 34/66) of patients did not recall receiving any information about signs of opioid toxicity (item J; Supplemental Appendix B) and interactions between opioids and alcohol (item K; Supplemental Appendix B).

## Discussion

This study found that the amount of opioids supplied after hospital discharge following surgery is generally more than necessary for patients’ postoperative pain management requirements. More than half of the patients still had some of their hospital supply of opioids unused within a month of discharge. This is consistent with the study from Victoria (*n* = 1450), which reported more than two thirds of their surgical patients had unused opioids at the two-week follow-up.^10^ Our study also found that 21% of patients who received opioids on discharge had not required any opioids in the 24 hours prior to discharge. This is consistent with the data from The Society of Hospital Pharmacists of Australia, showing that around 70% of Australian hospitals send patients home with opioids ‘just in case’.^19^ This ‘just in case’ prescribing shows that such patients may not need five days’ supply of opioids, and reducing their opioids supplied on discharge will minimise the amount of unused opioids in the community.

This study also showed that patients undergoing orthopaedic surgery were generally supplied more appropriate amounts of opioids on discharge compared to other specialities. In this study, 27% of patients undergoing orthopaedic surgery had ≥50% of their discharge opioids remaining after a month, which was significantly lower than other surgery types. These results appear consistent with another Australian study (*n* = 140) which involved patients undergoing an elective total knee or hip arthroplasty. Of these patients, 25% had >25% of their initial opioids supplied at discharge remaining.^20^ These results highlight that the amount of opioids supplied on discharge for patients who had total knee or hip arthroplasty are generally all used, while patients undergoing other surgeries may generally be supplied too many.

The results of this study also show that many patients were not being given optimal pain management advice, with only a few patients instructed to take simple non-opioid analgesics with their opioid in their discharge letter. Only half of the patients followed up were on regular paracetamol, and a minority were on regular NSAIDs. This is much lower than a previous Australian study, which found that 96% (*n* = 134) of patients who underwent orthopaedic surgery were on paracetamol and 11% were on NSAIDs on discharge.^19^ Regular use of paracetamol and NSAIDs with opioids postoperatively significantly reduces the oMEDD required 72 hours after a surgical procedure (82.7 ± 116.1 to 17.5 ± 46.0 mg, *P* < 0.001).^21^ Hence, the addition of a simple non-opioid analgesic has been recommended to reduce the usage of opioids and lower related side-effects,^22–24^ with additional efforts to ensure they are prescribed opioids on discharge after surgery.

The results of this study demonstrated that only around 36.4% of patients received a pain management plan in their discharge summary. Again, this result is inconsistent with another Australian study (*n* = 199). Of these patients, 86.1% had hospital discharge plan summaries sent to the general practitioners.^14^ Providing a pain management plan is currently recommended for all patients discharged with an opioid after surgery in the Australian therapeutic guidelines.^16^ An Australian study has shown that education of hospital staff and communication with general practitioners about opioid tapering plans can significantly increase the proportion of patients receiving opioid tapering plans (6.9–87.4%; *P* <0.001).^14^ We suggest that a similar intervention could be implemented in the study hospital to improve the proportion of patients receiving pain management plans in their discharge summary.

Most patients did not recall being informed about opioid side-effects, interactions between opioids and alcohol, or signs of opioid toxicity. Of these patients, 22.7% and 51.5% did not remember being made aware of opioid side-effects and toxicity, respectively. Providing take-home naloxone and education about opioid use and safety are recommended for patients who are at high risk of opioid toxicity.^17^ However, studies by Holland et al. and Penm et al. showed that healthcare professionals demonstrated a lack of training and education in take-home naloxone practice.^25,26^ An approach to ensure opioid side-effects and toxicity are discussed with all patients is required so that health professionals can identify those at high risk of opioid toxicity.^25,26^

Limitations of this study included a limited sample size due to recruitment being impacted by COVID-19. In addition, about 20% of patients were unable to be contacted for follow-up. This may have been attributed to the hospital number appearing as a private line on patients’ phones. To prevent this issue in the future, patients could be messaged on their phone before being called so they are aware of who is calling. Also, a dedicated phone with a listed number could be used so that it does not appear as a private number. Furthermore, a larger sample size from several health facilities would increase the generalisability of the results. Patients who did not respond to follow-up appeared to have a lower Charlson Comorbidity Index, highlighting that our results may not apply to healthier patients. In addition, opioid use and the number of tablets remaining were also self-reported and not verified by a third party. This may have resulted in response bias, such as social desirability bias, where patients may deny undesirable responses around opioid use and disposal.^27^ Also, the time difference in follow-up between patients of 7–28 days may have resulted in recall bias. In addition, the interview guide used was not tested for reliability or validity, except the questions based on SIMS. Lastly, only patients who had returned to the community were included in this study, which may have excluded highly complex patients discharged to rehabilitation facilities or nursing homes.

## Conclusions

Most patients are discharged from hospital with a five-day supply of opioids after surgery, and in the current study, around 40% of patients had more than half of their opioid supply remaining after they ceased taking their opioid. Most patients did not have a documented opioid management plan on their discharge summary. Although most patients recalled receiving information about their opioids, more than half did not recall receiving any information about the signs of opioid toxicity or interactions between opioids and alcohol. The Australian Commission on Safety and Quality in Health Care recently released the Opioid Analgesic Stewardship in Acute Pain Clinical Care Standard to improve opioid use in hospital and on discharge.^28^ However, the best way to implement the Standard is unknown currently.

## Supplemental Material

sj-pdf-1-aic-10.1177_0310057X231163890 - Supplemental material for Supply of opioids and information provided to patients after surgery in an Australian hospital: A cross-sectional study Click here for additional data file. Supplemental material, sj-pdf-1-aic-10.1177_0310057X231163890 for Supply of opioids and information provided to patients after surgery in an Australian hospital: A cross-sectional study by Ian SH Fong, Chin Hang Yiu, Matthew D Abelev, Sara Allaf, David A Begley, Bernadette A Bugeja, Kok Eng Khor, Joanne Rimington and Jonathan Penm in Anaesthesia and Intensive Care
