# Supplementary material for: Supply of opioids and information provided to patients after surgery in an Australian hospital: A cross-sectional study
Source: Anaesth Intensive Care. 2023 Jun 26;51(5):340–7. doi: 10.1177/0310057X231163890 (PMC10493037; doi:10.1177/0310057X231163890)
Supplement: sj-pdf-1-aic-10.1177_0310057X231163890 - Supplemental material for Supply of opioids and information provided to patients after surgery in an Australian hospital: A cross-sectional study [file sj-pdf-1-aic-10.1177_0310057X231163890.pdf]

Supplementary Material

APPENDIX A: Baseline data collection form

**Patient information**	
Sex:	Age:
**Type of surgery**	
Specialty:<br>Surgery is: ☐$_1$ Elective ☐$_2$ Emergency	
Opioid history (according to electronic medical record):	☐$_1$ No opioids used 2 weeks prior to admission<br>☐$_2$ Only used opioids 'as needed' 2 weeks prior to admission<br>☐$_3$ Regular opioid(s) used prior to admission for any indication (e.g. slow-release tablets, patches or regular oxycodone)
**Opioid use 24 hours prior to discharge**	
Name, dose and frequency of opioids taken:	
Total dose of each regular opioid taken:<br>Total dose of each 'as needed' opioid taken:	
Total dose of all opioids taken (oMEDD over 24 hour period):	
**Opioids provided on discharge**	
Name, dose and frequency of opioids prescribed by doctor:	
Daily dose of all regular opioids prescribed in oMEDD:<br>Daily dose of each 'as needed' opioid prescribed in oMEDD:	
Quantity of each opioid prescribed by doctor:<br><br>Number of days for each:	
Non-opioid analgesics prescribed: ☐$_1$ paracetamol<br>☐$_2$ NSAID (specify) ________________	☐$_4$ regular ☐$_5$ as needed

☐3 Other (specify)__________________	☐6 regular ☐7 as needed<br>☐8 regular ☐9 as needed

**Plan with opioids on discharge**
Plan documented in discharge summary (tick all that apply): ☐1 Review by GP<br>☐2 Wean dose<br>☐3 Cease after _____ days (specify number)<br>☐4 None mentioned<br>☐5 No discharge summary available

oMEDD = oral Morphine Equivalent Daily Dose

APPENDIX B: Phone interview questionnaire and data collection form.

**Patient's current usage of opioids**	
Which opioid medicines are you still taking?	☐1 Still taking (specify all): ________________<br>____<br>☐2 Not taking (specify all):<br>_________________<br>____
For <insert opioid medicine(s) you are **still taking**>, who made that decision?	☐1 Self<br>☐2 Doctor<br>☐3 Pharmacist
For <insert opioid medicine(s) you are **not taking**>, who made that decision?	☐1 Self<br>☐2 Doctor<br>☐3 Pharmacist
Do you have any other comments about your use of opioid medicines?	
**Patient's current stock of opioids**	
Do you have any hospital supply of <insert names of all opioid medicines prescribed to participant> remaining?	☐1 Yes (specify all):<br>______________________<br>____<br>☐2 No (specify all):<br>_______________________<br>____
If yes, how many tablets or how much volume is left?	
If no, did you finish taking them, or dispose of them?	☐1 Finished taking<br>☐2 Disposed

	☐3 Other ________________
If they were disposed, how did you dispose of them?	
**Patient's review with the GP**	
Have you been seen by the GP following your discharge?	☐1 Yes<br>☐2 No
Was there a discussion about your pain management?	☐1 Yes<br>☐2 No
Were you given another prescription for opioid medicines,<br>or any other pain reliever medicine?	☐1 Yes (specify name, strength and frequency)<br>____<br>☐2 No
If yes, how many tablets did you receive?	
**Patient's provision of opioid information**	
Were you given any information about your opioid medicines on discharge?	☐1 Yes<br>☐2 No
If yes, by who? (Tick all that apply)	☐1 Pharmacist<br>☐2 Doctor<br>☐3 Nurse
If yes, what form of information? (Tick all that apply)	☐1 Verbal counselling<br>☐2 Written information
If yes, have you ever had to refer or think back to the information provided?	☐1 Yes<br>☐2 No
**Patient's satisfaction of opioid information provided (adapted from SIMS)**	
We would like to ask how satisfied you were with the information you received about your opioid medicines while you were in hospital. There are twelve items of information, and I will state them one at a time. For each item, please rate your satisfaction by letting me know whether you received too much information, too little information, about the right amount of information, none was received, or if none was needed.	

		Too much	About right	Too little	None received	None needed
A	What your medicines are for	☐1	☐2	☐3	☐4	☐5
B	How long they take to act	☐1	☐2	☐3	☐4	☐5
C	How long you will need to be on them for	☐1	☐2	☐3	☐4	☐5
D	How to use them	☐1	☐2	☐3	☐4	☐5
E	How to get a further supply	☐1	☐2	☐3	☐4	☐5
F	Whether the medicine has any unwanted effects (side effects)	☐1	☐2	☐3	☐4	☐5
G	What are the chances of getting side effects	☐1	☐2	☐3	☐4	☐5
H	What you should do if you get unwanted side effects	☐1	☐2	☐3	☐4	☐5
I	Information specifically about constipation and how to prevent it whilst taking opioid medicines	☐1	☐2	☐3	☐4	☐5
J	Information about signs that you are taking too many opioid medicines, such as:<br>- difficulty staying awake or being woken up<br>- breathing is more difficult, shallow or slower	☐1	☐2	☐3	☐4	☐5
K	Whether you can drink alcohol whilst taking them	☐1	☐2	☐3	☐4	☐5
L	Whether any of the medicines interfere with each other	☐1	☐2	☐3	☐4	☐5

What other information about your opioid medicines would you have also liked to receive while in hospital?
